# A pilot feasibility and acceptability trial of an internet indicated prevention program for perfectionism to reduce eating disorder symptoms in adolescents

**DOI:** 10.1007/s40519-024-01654-8

**Published:** 2024-04-12

**Authors:** Amy O’Brien, Rebecca Anderson, Trevor G. Mazzucchelli, Sarah Ure, Sarah J. Egan

**Affiliations:** 1 Discipline of Psychology, School of Population Health, Curtin University, Perth, Australia; 2 Faculty of Health Sciences, enAble Institute and School of Population Health, Curtin University, GPO Box U1987, Perth, WA 6845 Australia

**Keywords:** Eating disorder, Perfectionism, Co-design, Adolescent, Intervention, Prevention

## Abstract

**Purpose:**

Perfectionism is a transdiagnostic risk factor for eating disorders. Treating perfectionism can reduce symptoms of eating disorders. No research has examined an indicated prevention trial using internet-based Cognitive-Behavioural Therapy for Perfectionism (ICBT-P) in adolescent girls at elevated risk for eating disorders. Our aim was to conduct a preliminary feasibility trial using a co-designed ICBT-P intervention. It was hypothesised that a higher proportion of participants in the ICBT-P condition would achieve reliable and clinically significant change on perfectionism, eating disorders, anxiety and depression, compared to waitlist control.

**Methods:**

Twenty-one adolescent girls with elevated symptoms of eating disorders (*M* age = 16.14 years) were randomised to a 4-week online feasibility trial of a co-designed ICBT-P prevention program or waitlist control. Qualitative surveys were used to gain participant perspectives.

**Results:**

The ICBT-P condition had a higher proportion of participants achieve reliable change and classified as recovered on perfectionism and symptoms of eating disorders and anxiety, compared to waitlist control. Qualitative findings indicated that 100% of participants found the program helpful.

**Conclusion:**

The results indicate ICBT-P is a feasible and acceptable program for adolescent girls with elevated eating disorder symptoms. Future research is required to examine outcomes in a randomised controlled trial.

**Level of evidence:**

Level III: Evidence obtained from well-designed cohort or case–control analytic studies.

***Trial registration number*:**

This trial was prospectively registered with Australian and New Zealand Clinical Trials Registry (ACTRN12620000951954P) on 23/09/2020.

**Supplementary Information:**

The online version contains supplementary material available at 10.1007/s40519-024-01654-8.

The typical age of onset for eating disorders is adolescence [1]. One risk factor for eating disorders is perfectionism [2]. Clinical perfectionism is self-esteem based on achievement, despite negative consequences [3]. Perfectionism is significantly associated with symptoms of eating disorders in children, adolescents and adults [4–7]. Meta-analytic reviews have demonstrated that cognitive behaviour therapy for perfectionism (CBT-P) achieves medium-to-large effect size reductions in perfectionism and symptoms of eating disorders, anxiety and depression [8–10].

Internet-based CBT-P (ICBT-P) has evidence of efficacy in both guided and unguided formats [11–15]. ICBT-P is not only efficacious as an intervention program for eating disorders, but also as a prevention [5, 10]. Universal prevention is applied to whole populations, selective prevention for individuals at risk, and indicated prevention for those with heightened eating disorder symptoms [16]. Shu et al. [15] conducted a selective prevention trial of unguided ICBT-P with 94 female adolescents and found compared to an active treatment and also waitlist control, significant reductions in eating disorder, depressive and anxious symptoms at post-test, maintained at six months follow-up. To date, no indicated prevention trials using ICBT-P have been conducted with adolescent girls at elevated risk for disordered eating.

There have also been few qualitative studies exploring participant experiences of online therapies for eating disorders, and none specifically have examined qualitative feedback on ICBT-P as an indicated eating disorder prevention program. Only two qualitative studies to date have conducted an exploration of participant experiences of ICBT-P [17, 18]. Rozental et al. [17] conducted a study with adults from a 12-week trial of guided ICBT-P [12]. While many participants expressed positive comments about the acceptability of ICBT-P, some expressed a desire for face-to-face contact [19]. Egan, Neal [18] conducted a feasibility and acceptability trial of a parent-supported version of ICBT-P co-designed with parents for adolescents with eating disorders. Feedback was positive, with 100% of parents and adolescents reporting ICBT-P useful, however some participants desired a more interactive online approach to the PDF format used [18].

There have been no studies of ICBT-P as an indicated prevention program for adolescents at an elevated risk for eating disorders. To improve outcomes, it is important that effective prevention programs exist, and that they consider adolescent experiences of ICBT-P to better cater to young people’s preferences and needs. Co-designed research incorporates the ideas and perspectives of the target demographic in the development and design of the intervention [20]. Interventions which are co-designed are aimed at ensuring that the voices of young people are heard throughout the process of design, delivery and refinement of interventions and a key method to ensure an intervention is relevant to those it aims to help [20]. It has been argued that co-design is critical to enhance the quality and delivery of interventions by engaging together with individuals with lived experience to improve the relevance and uptake of interventions [18]. Co-design of online interventions for individuals with eating disorders has received limited attention, with most studies involving individuals with lived experience at the planning stage [21]. A systematic review in 2021 identifying only eight co-designed studies, with the authors concluding meaningful co-design should be increased throughout the entire research process in eating disorder interventions [21]. The co-design of online interventions should involve collaboration on design and refinement of an intervention [22]. It has been recommended that online interventions for eating disorders consider consumer needs, preferences and feedback to address low rates of uptake and engagement [23].

Given the outlined importance of co-design of interventions, the aim of the current study was to examine the feasibility and acceptability of a co-designed ICBT-P program delivered as an interactive website intervention, for female adolescents with elevated eating disorder symptoms. A pilot feasibility and acceptability trial was conducted as a larger participant sample would as required to conduct a randomised controlled trial (RCT) than was available in the timeframe of this study. It is hypothesised that a significantly higher proportion of participants in the ICBT-P condition will demonstrate post-test reliable change on measures of perfectionism, eating disorders, depression and anxiety than those in the control condition. In addition, to inform whether the intervention is acceptable, this study aimed to use qualitative methodology to answer the question “What can be learned from female adolescents’ experiences completing a co-designed ICBT-P program?”.

## Methods

### Intervention development

The ICBT-P intervention was developed based on the ‘Overcoming Perfectionism’ self-help guide by Shafran et al. [24]. The online intervention consisted of eight modules, plus a brief introductory module, with the aim of reducing clinical perfectionism. The intervention was delivered on the website ‘www.youthperfectionism.org’, available at www.overcomingperfectionism.com, and was co-designed together with a Youth Advisory Group of adolescents with lived experience of perfectionism (for details of the co-design of the intervention, see O’Brien et al. [25]).

### Participants

Inclusion criteria were: (i) identifying as female; (ii) age 13 to 18 years; (iii) internet access; (iv) access to a general practitioner; and (v) elevated eating disorder symptoms. Exclusion criteria were: (i) a moderate or high suicide risk and (ii) currently receiving psychotherapy. Participants could continue taking any psychotropic medications, but were requested to inform if there were any changes to their medication during the trial.

### Recruitment

Participants were recruited through free and paid social media advertising, promotion of the study to Western Australian (WA) Independent High Schools, and recruitment emails and social media posts by eating disorder advocacy and treatment services. Social media advertisements were placed across Facebook and Instagram, with adverts targeting both adolescents and parents of female adolescents. High school principals, wellbeing coordinators and school psychologists at coeducational and female-only WA Independent Schools were also provided recruitment materials.

**Fig. 1:**
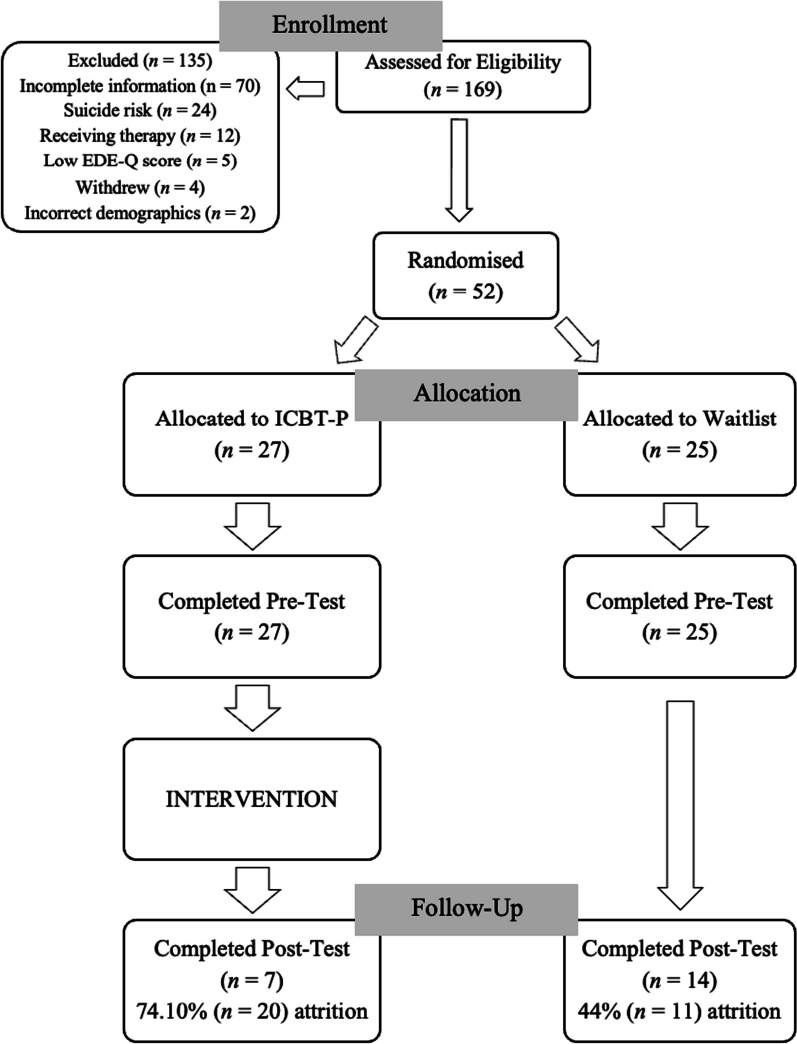
CONSORT flowchart of participant recruitment and retention

Figure 1 depicts a flowchart of participant recruitment and retention, demonstrating that 79.88% of prospective participants were ineligible. Of those screened out, 17.78% were due to reporting a moderate to high risk for suicide. Group allocation was randomised with a method of simple random allocation.

There were 52 participants (mean age = 16.36 years (*SD* = 1.19, range 13–18 years, 100% female). There was no significant difference in age across groups at allocation (experimental *M* = 16.22, *SD* = 1.28, waitlist *M* = 16.52, *SD* = 1.08; *t*(50) = − 0.90, *p* = 0.372, two-tailed). At post-assessment, 40.38% completed follow-up measures, however this was not proportionate between groups, with two-thirds of completed measures in the waitlist condition. The post-test follow-up consisted of 21 individuals aged 13 to 18 (*M* = 16.14, *SD* = 1.39; see Table 1).

**Table 1:** Means and standard deviations reported by group at pre- and post-test for age and clinical outcome variables, and group equivalence testing at pre-test

Variable	ICBT-P		Waitlist control		Group equivalence testing at pre-treatment (two-tailed)
	M (SD)		M (SD)		
	Pre-treatment (*n* = 27)	Post-treatment (*n* = 7)	Pre-treatment (*n* = 25)	Post-test (*n* = 14)	
Age	16.22 (1.28)	16.29 (1.80)	16.52 (1.08)	16.07 (1.21)	*t*(50) = − .90, *p* = .372
EDE-Q global	3.20 (1.23)	1.84 (0.57)	3.37 (1.41)	3.10 (1.30)	*t*(50) = − .46, *p* = .650
CPQ	32.15 (6.27)	27.57 (5.86)	34.72 (6.51)	35.01 (5.37)	*t*(50) = − 1.45, *p* = .153
RCADS Anxiety	63.70 (12.79)	58.29 (9.48)	65.84 (13.00)	67.00 (12.27)	*t*(50) = − .60, *p* = .553
RCADS Depression	63.70 (12.57)	59.14 (8.57)	64.60 (15.42)	65.36 (15.10)	*t*(50) = − .23, *p* = .819

ICBT-P = Internet-Based Cognitive Behaviour Therapy for Perfectionism; EDE-Q Global = Global score on the Eating Disorder Examination Questionnaire; CPQ = total score on CPQ (12-Item version); RCADS Depression = Revised Child Anxiety and Depression Scales (RCADS) T-score on the Depression subscale, RCADS Anxiety = T-score on the total subscales for anxiety

A total of five responses were received from ICBT-P participants on the qualitative survey (*M* = 15.80, *SD* = 1.92, range 13–17 years). This represented 71.43% of participants who completed the program (18.52% of overall participants allocated to the intervention).

### Measures

#### Clinical Perfectionism Questionnaire (CPQ) [26]

The CPQ is a 12-item measure of clinical perfectionism with good internal consistency and concurrent validity [27–31].

#### Revised Children’s Anxiety and Depression Scale (RCADS) [32]

The RCADS is a 47-item measure of anxiety and depression in children and adolescents [33]. The RCADS measures anxious and depressive symptoms across six subscales with acceptable internal consistency (subscales; 0.78 to 0.88 [32]).

#### Eating Disorder Examination Questionnaire (EDE-Q; [31]

The EDE-Q is a 28-item self-report measure of eating disorder symptoms [34, 35] with good reliability and validity [36, 37] including in adolescents [15, 38, 39]. An EDE-Q global score of ≥ 1.5 in a community sample indicates elevated symptoms of an eating disorder as per normative data across paediatric and adult clinical and community samples which indicate scores of > 1.5 being the approximate cut-off for elevated symptoms of disordered eating [34, 36, 40].

#### Mini-international neuropsychiatric interview for children and adolescents (MINI-Kid) module B1 [41]

The MINI-KID six items assess suicidal ideation and behaviour over the past month, and lifetime suicide attempts [41]. Scores above five = moderate risk and greater than 10 = high risk. The MINI-KID was administered from October 2021 and discontinued in September 2022 due to the high rate of people who were being screened out.

#### Columbia Suicide Severity Rating Scale (C-SSRS) [42]

The C-SSRS assesses current suicide risk in adolescents and adults [42]. Five items were used from September 2022 to trial end in December 2022, which assessed current suicidal ideation and behaviours. It has good internal consistency and sensitivity [42]. The C-SSRS replaced the MINI-KID which was overly sensitive in ruling out participants as it did not distinguish between previous and current suicide risk.

#### Adherence

Participants were asked to complete a brief four-item questionnaire [43] at the completion of each module to assess adherence with the intervention protocol. The questions measured engagement with the intervention and included items such as “how much of the module did you complete?”, where participants provided estimated percentage ratings (0%, 25%, 75%, 100%), time spent on the module (0 min, 1–30 min, 30–60 min, 60–120 min, 120 + minutes), usefulness (1 = not useful at all to 5 = extremely useful) and ease of use (1 = strongly disagree to 5 = strongly agree).

#### Qualitative survey

A qualitative survey of 10 open-ended questions was provided to ICBT-P participants at post-treatment to gain feedback on the intervention (see supplementary materials Table S1 for questions).

### Procedure

This study was approved by the Curtin University HREC (approval number: HRE2020-0626) and was prospectively registered with the Australian and New Zealand Clinical Trials Registry (approval number: ACTRN12620000951954P). Adolescents and their parents were directed to the www.youthperfectionism.org website to complete digital information and consent forms. Participants who completed consent forms were then screened online for inclusion/exclusion criteria.

Participants were randomly allocated to ICBT-P intervention or waitlist control, and recommended to complete two modules per week over four weeks. Generic email reminders to complete the intervention were sent weekly. Participants were emailed a link four weeks after initial program access and requested to complete post-intervention measures.

#### Data analysis

Initially the trial was planned to be an RCT (see published protocol; [25]), however changed to a feasibility trial due to low numbers of accepted participants and the limited timeframe available within the context of the research being conducted as a dissertation of the first author. Data were analysed without a focus on statistical significance, and in accordance with the clinical significance and reliable change (Jacobson and Truax [44, 45]; for further details see supplementary materials). Fisher’s exact test (1-sided) was used to establish whether the intervention group had a significantly higher proportion of participants classified as recovered compared to waitlist control [46].

Inductive thematic analysis was used for qualitative responses. The six phases of thematic analysis of Braun and Clarke [47] were used as a guide. In summary, these steps included developing an initial overview of the data, generating initial codes, deciding on what themes may be and generating a draft of potential themes, followed by defining and naming themes [47]. AO conducted the coding of themes manually, with SE providing consensus on codes via discussion about the content of the themes. Ongoing review of the themes through numerous discussions occurred until the final themes were reached through mutual agreement between AO and SE. Given the importance of positionality of researchers in qualitative analysis [47], we reflected on our backgrounds. AO identifies as a cisgender woman from Australia who is a Clinical Psychologist, having worked with adolescents and is an early career researcher in the area of perfectionism and eating disorders. SE is also a Clinical Psychologist who identifies as a cisgender woman from Australia, who has many years’ experience as researcher in perfectionism and eating disorders.

## Results

As seen in Table 1, the groups were equivalent on all variables at pre-treatment. Most participants (81.48%) completed module 1, 40.74% module 2, 29.63% module 3, 22.22% module 4, 14.81% modules 5 and 6, 7.41% module 7 and 3.70% module 8. Modules were unlocked and participants could move between them, and non-completion of the module adherence check did not prevent participants from progressing to the next module. Hence, initial uptake for the intervention was good, with 81.48% of ICBT-P participants completing the first module adherence check. Ongoing use showed a sharp decline from Module 2 onwards, with only one individual completing the adherence check by Module 8. However, as seven individuals completed the ICBT-P program and post-intervention measures, this would indicate that at least seven participants did complete the program, but chose to not report adherence checks.

Table 2 indicates those in the ICBT-P intervention group experienced greater reliable change and decreases in symptoms than waitlist control. No participant in the ICBT-P condition deteriorated across any measures. In waitlist control, there were nine occasions across where a participant experienced a deterioration and four occasions when a participant did achieve a reliable decrease in an outcome measure, however the predominant finding was that no change occurred during waitlist control.

**Table 2:** Participant *n* and % reported by condition who experienced reliable change

Variable	ICBT-P			Waitlist control		
	*↓ n, %*	*↑ n, %*	*␀ n, %↓ n, %*	*↓ n, %*	*↑ n, %*	*␀ n, %*
EDE-Q global	6 (85.71%)	0 (0%)	1 (14.29%)	3 (21.43%)	4 (28.57%)	7 (50%)
CPQ total	4 (57.14%)	0 (0%)	3 (42.86%)	0 (0%)	0 (0%)	14 (100%)
RCADS Anxiety	5 (71.43%)	0 (0%)	2 (28.57%)	1 (7.14%)	3 (21.43%)	10 (71.43%)
RCADS Depression	2 (28.47%)	0 (0%)	5 (71.43%)	0 (0%)	2 (14.29%)	12 (85.71%)

*N* = 21 (ICBT-P *n* = 7, Waitlist control *n* = 14); ↓ n, % = the number and percentage of participants who experienced reliable change constituting a decrease in the respective outcome measure; ↑ n, % = the number and percentage of participants who experienced reliable change constituting an increase in the respective outcome measure; ␀ n, % = the number and percentage of participants who did not experience any reliable change on the respective outcome measure; ICBT-P = Internet-Based Cognitive Behaviour Therapy for Perfectionism; EDE-Q global = Global score on the Eating Disorder Examination Questionnaire; CPQ total = total score on the 12-item version of the Clinical Perfectionism Questionnaire (CPQ); RCADS Depression = Revised Child Anxiety and Depression Scales (RCADS) T-score on the Depression subscale, RCADS Anxiety = T-score on the total subscales for anxiety

Table 3 provides an overview clinical classification at post-test. Fisher’s exact test (1-sided) indicates a significantly higher proportion of participants in ICBT-P were deemed recovered on eating disorder symptoms, compared to control (*p* = 0.017). A significantly higher proportion of participants in the ICBT-P group achieved a classification of recovered on perfectionism (*p* = 0.006) compared to control. Conversely, a significantly higher proportion of participants in waitlist control were classified as no change (*p* = 0.006) when compared to ICBT-P. A higher proportion of participants in ICBT-P also achieved a rating of recovered on anxiety (*p* = 0.006) compared to control. On the EDE-Q, 71.43% of ICBT-P participants were recovered, and 14.29% improved. As per Fisher’s test, these findings were significantly better than waitlist control, where 50% experienced no change in EDE-Q symptoms, and four participants experienced deterioration. There was a significant difference on perfectionism (*p* = 0.006), with 57.14% of ICBT-P participants meeting criteria for recovery, and no participants in control, where all waitlist participants had no change. In relation to anxiety, 71.43% of intervention participants met criteria for improved or recovered. Whilst only five ICBT-P participants had scores suggesting reliable change, all participants had a decrease in anxiety and met criteria for the functional or subclinical range (see supplementary materials Table S2). Five ICBT-P participants had scores in the clinical range at pre-assessment, with no participants meeting clinical thresholds at post-intervention. In waitlist control, three participants deteriorated at post-test, the majority (71.43%) showed no change. On depression, two participants in ICBT-P met criteria for recovered, with five participants classified as no change. Three participants who were in the clinical range at pre-test achieved scores in the functional range at post-test (see supplementary materials Table S2). Waitlist control participants predominately had no change, although two deteriorated.

**Table 3:** Number and percentage of participants by group meeting criteria for reliable and clinically significant change at post-intervention

Outcome		ICBT-P		Waitlist Control		Fisher's exact test
		*n*	%	*n*	%	(1-sided)
EDE-Q global						
	Recovered	5	71.43	2	14.29	*p* = .017*
	Improved	1	14.29	1	7.14	*p* = .567
	No change	1	14.29	7	50	*p* = .133
	Deteriorated	0	0	4	28.57	*p* = .167
CPQ total						
	Recovered	4	57.14	0	0	*p* = .006**
	Improved	0	0	0	0	*p* = 1.00
	No change	3	42.86	14	100	*p* = .006**
	Deteriorated	0	0	0	0	*p* = 1.00
RCADS Depression						
	Recovered	2	28.57	0	0	*p* = .100
	Improved	0	0	0	0	*p* = 1.00
	No change	5	71.43	12	85.71	*p* = .407
	Deteriorated	0	0	2	14.29	*p* = .433
RCADS Anxiety						
	Recovered	4	57.14	0	0	*p* = .006**
	Improved	1	14.29	1	7.14	*p* = .567
	No change	2	28.57	10	71.43	*p* = .080
	Deteriorated	0	0	3	21.43	*p* = .274

*N* = 21 (ICBT-P *n* = 7, Waitlist control *n* = 14). ICBT-P = Internet-Based Cognitive Behaviour Therapy for Perfectionism; EDE-Q global = global score on the Eating Disorder Examination Questionnaire; CPQ 12-Item total = Total score on the 12-Item version of the Clinical Perfectionism Questionnaire (CPQ); RCADS Depression = Revised Child Anxiety and Depression Scales (RCADS) T-score on the Depression subscale, RCADS Anxiety = T-score on the total subscales for anxiety

* *p* < .05 ** *p* < .01 *** *p* < .001

### Qualitative findings

After thematic analysis, nine overarching themes were formulated.

#### Contributing to research

One theme appeared to be a desire to participate in research. As one young person said, *“I wanted to be a part of the program in hopes… of contributing to greater research to help others who feel similar things”.* This sentiment underscored that young people want to engage in research and to help others in the future going through a similar experience.

#### Wanting to understand or improve oneself

The predominant theme for motivation to engage related to young people’s desire to seek help. Young people said perfectionism was holding them back from achieving their goals, for example *“Perfectionism was getting in the way of my daily tasks and making me feel bad about myself”.* Young people outlined a desire to understand themselves and improve wellbeing.

#### ICBT-P as a helpful program for adolescents

The feedback on ICBT-P was overwhelmingly positive (n = 5, 100%), with young people reporting the program was helpful, they liked the content and design, and had noticed improvements. All ICBT-P participants would recommend the program to others:

*I think the program was useful in identifying why perfectionism is unhealthy at times and provided a few coping strategies for these situations.*

#### The importance of relatability

Many young people highlighted how strongly they related to the content, and discussed how this made the program seem more relevant and personalised. One young person stated, *“every issue raised in the program was relatable and I felt like it was talking to me personally”.* Another said, *“I felt that I related to a lot of the examples throughout the program, which helped me understand that I’m not alone”.*

#### Privacy and anonymity

One of the benefits young people noted was the anonymity and privacy internet treatment afforded them. Several young people stated anxiety and concern over a therapists’ judgement as a reason that made online treatment appealing, with one young person saying they *“didn’t have to admit flaws to people”.*

#### Interactivity

Most feedback regarding improvements to the program related to interactivity. While one young person stated, *“I loved how interactive it was,”* another wanted, *“More print outs- writing by hand often makes you think and remember more than typing”.*

#### Flexibility

One of the key reasons young people voiced an interest in online therapy was flexibility; *“I liked that you could complete it at your own pace, and at home… I also didn’t feel pressured to complete tasks since they could be done at any time through the week (there weren’t strict due dates).”* Young people acknowledged online therapy may not be right for everyone, but it was important to have choice:

*I think that the effectiveness of online therapy varies based on the person. Personally, I enjoyed this program as I could do it at home, away from people.*

#### Optimal social support for online therapy

Despite the desire for anonymity, some young people raised connecting with others could be an additional support through guidance available. One young person identified an idea of peer-support:

*I think it would be beneficial to be able to meet with other people completing the program or other people that also experience perfectionism to get more personal experience.*

#### Barriers to engagement

Young people identified various barriers to engagement, for example *“Sometimes I’d forget to do the program because I had to motivate myself to do it and sometimes I didn’t want to”.* Another said the behavioural experiments were too lengthy, but felt they were important, *“I did think that some of the activities were a little bit excessive, e.g., conducting experiments. They didn’t prove to be as effective for me, but I am still glad that I did them”.*

## Discussion

This was the first examination of ICBT-P as an indicated prevention for adolescents at an elevated risk for eating disorders. The preliminary results indicated a greater proportion of participants in ICBT-P intervention demonstrated reliable reductions in perfectionism and symptoms of eating disorders and anxiety compared to waitlist control. There was no difference between intervention and control on depression, however floor effects may have impacted, with low symptoms of depression at pre-intervention.

Given the small sample size and high attrition, results must be interpreted with caution. However, preliminary results indicate participants at pre-intervention with extremely elevated eating disorder symptoms had scores in the functional range at post-intervention. The decreases in eating disorder symptoms are in line with Shu et al.’s [15] ICBT-P selective prevention trial, who also found larger effects on eating disorder symptoms than anxiety and depression. Our results are also comparable to a recent feasibility trial by Egan, Neal [18] of co-designed parent-supported ICBT-P for eating disorders, which found adolescents moved from a clinical range pre-intervention to sub-clinical post-intervention [18].

Harris et al. [48] state patient-satisfaction should be one of the ultimate goals of treatment. All participants in the intervention said they would recommend the program to a friend or family member and found the program helpful and relatable. Young people reported valuing the anonymity and flexibility of an unguided internet intervention, and accessing therapy without feeling judged, consistent with previous research [17, 18, 49, 50]. Also consistent with past research, treating perfectionism reduced symptoms of eating disorders [9, 10] despite never explicitly addressing the topic of disordered eating. Research in eating disorder interventions has suggested that individuals prefer broader programs (79% of participants) rather than programs highly specific to their presenting symptoms [51], supporting the acceptability of a perfectionism program for eating disorders.

### Strengths and limits

A strength was the co-design of the intervention with lived experience experts. Additionally, although the program was unguided, young people expressed a high degree of relatability to the point where they reported feeling the intervention was talking to them personally. This relatability may have helped overcome the potential for online therapies to be perceived as impersonal or lacking a human element [52]. Our findings support the importance of co-designed interventions and how this can improve outcomes, consistent with recent research on co-designed ICBT-P [53]

A significant limitation was the high rate of attrition, most of which occurred prior to allocation, meaning the RCT planned was turned into a feasibility study. The small sample size also resulted in variables that may help to understand uptake and dropout not being able to be statistically examined. In a future RCT of ICBT-P as an indicated prevention for eating disorders, the variables which predict uptake and dropout should be examined. The requirement for parental consent for participants younger than 15 years of age likely discouraged participation. Consistent with previous literature [54, 55], adolescents expressed a preference for completing internet interventions independently without parent involvement. Future research should examine the number of modules that participants complete. As the intervention program was unguided and modules unlocked to allow participants to move through them at will, it is impossible to say with certainty at what stage individuals may have dropped out of the study during the intervention phase. Although participants were requested to complete an adherence to treatment survey following completion of each module, modules could not be locked to ensure this was completed prior to advancing in the program. Qualitative feedback indicated at least some ICBT-P participants completed more modules than the adherence data would suggest. Future research should implement measures of uptake, usage and adherence to further understand participant engagement and feasibility.

In summary, ICBT-P may be a feasible indicated prevention program for adolescent girls at an increased risk for eating disorders. Future RCTs should prioritise strategies that will increase participant retention and seek to obtain long-term follow-up to understand prevention effects of the intervention.

### Supplementary Information

Below is the link to the electronic supplementary material.

Supplementary file1 (DOCX 27 KB)

## Data Availability

The datasets generated and/or analysed during the current study are not publicly available due to ethical considerations, but are available from the corresponding author on reasonable request and subject to Institutional approvals.
