# Supplementary material for: A pilot feasibility and acceptability trial of an internet indicated prevention program for perfectionism to reduce eating disorder symptoms in adolescents
Source: Eat Weight Disord. 2024 Apr 12;29(1):27. doi: 10.1007/s40519-024-01654-8 (PMC11009734; doi:10.1007/s40519-024-01654-8)
Supplement: Supplementary file 1 — Supplementary file1 (DOCX 27 KB) [file 40519_2024_1654_MOESM1_ESM.docx]

**Supplementary Table S1 – Qualitative Survey**

*Qualitative Survey Questions for Intervention Participants at Post-Treatment*

*___________________________________________________________________________*

1. What made you decide to participate in this online treatment program?
2. Can you please tell me how relevant and useful this program was to your own experiences of symptoms of perfectionism and eating problems?
3. What did you like about this program?
4. What, if any, were the positive parts of completing a treatment program online?
5. What would you change to improve this program?
6. Were there particular aspects of the program that you wanted less of?
7. How do you feel about online therapy or treatment for mental health problems?
8. Would you have preferred to have some guidance in using this program, or would you prefer working through it on your own like you did?
9. Would you recommend this program to your friends or family members?
10. Any other comments, feedback, or suggestions you would like to make?

___________________________________________________________________________

**Reliable And Clinically Significant Change Categorisation**

In line with Jacobson and Truax [1] and Hageman and Arrindell [2] participants were considered “recovered” if the RC is reliable, and if their post-treatment score moved closer to the functional mean and exceeded the clinical threshold for clinical cut-offs [2]. A participant was classified as “improved” if the RC was reliable, but their post-test score was still within a range of a dysfunctional population [2]. Participants were classified as “deteriorated” if reliable change was deemed to have occurred and their post-test score has moved further into the dysfunctional range, and as “unchanged” if no reliable change occurred [2]. Data generated in Shu, O’Brien et al. [3] for the CPQ, EDE-Q, and RCADS Depression and Anxiety scales was used for normative data to calculate reliable change (RC). This study [3] used a sample highly comparable with the current study; a community sample of 267 Australian female adolescents aged 14 – 19 years of age.

1. Jacobson NS, Truax P. Clinical significance: A statistical approach to defining meaningful change in psychotherapy research. Journal of Consulting and Clinical Psychology. 1991;59(1):12-9. <https://doi.org/10.1037/0022-006X.59.1.12>

2. Hageman WJ, Arrindell WA. Establishing clinically significant change: Increment of precision and the distinction between individual and group level analysis. Behaviour Research and Therapy. 1999;37:1169-93. <https://doi.org/10.1016/S0005-7967(99)00032-7>

3. Shu CY, O’Brien A, Watson HJ, Anderson RA, Wade TD, Kane RT, et al. Structure and validity of the Clinical Perfectionism Questionnaire in female adolescents. Behavioural and Cognitive Psychotherapy. 2020;48(3):268-79. <https://doi.org/10.1017/S1352465819000729>

| **Supplementary Table S2 – Pre-Treatment and Post-Treatment Score Classification Reported for Each Participant on the EDE-Q, CPQ, and RCADS** | | | | | | | | | |
| --- | --- | --- | --- | --- | --- | --- | --- | --- | --- |
|  | EDE-Q Global | | | |  | CPQ | | | |
| Group | Pre-Treatment Score Classification | Post-Treatment Score Classification | Reliable Change Achieved | Clinically Significant Classification |  | Pre- Treatment Score Classification | Post-Treatment Score Classification | Reliable Change Achieved | Clinically Significant Classification |
| ICBT-P P1 | Elevated, functional range | Functional | No | Unchanged |  | Functional | Functional | No | Unchanged |
| ICBT-P P2 | Extremely elevated, clinical range | Functional | Yes | Recovered |  | Functional | Functional | Yes | Recovered |
| ICBT-P P3 | Clinical range | Functional | Yes | Recovered |  | Functional | Functional | Yes | Recovered |
| ICBT-P P4 | Extremely elevated, clinical range | Elevated, clinical range | Yes | Improved |  | Functional | Functional | No | Unchanged |
| ICBT-P P5 | Extremely elevated, clinical range | Functional | Yes | Recovered |  | Functional | Functional | Yes | Recovered |
| ICBT-P P6 | Elevated, clinical range | Functional | Yes | Recovered |  | Clinical | Functional | No | Unchanged |
| ICBT-P P7 | Extremely elevated, clinical range | Functional | Yes | Recovered |  | Functional | Functional | Yes | Recovered |
|  |  |  |  |  |  |  |  |  |  |
| Waitlist P8 | Extremely elevated, clinical range | Extremely elevated, clinical range | No | Unchanged |  | Clinical | Clinical | No | Unchanged |
| Waitlist P9 | Clinical range | Functional | Yes | Recovered |  | Functional | Functional | No | Unchanged |
| Waitlist P10 | Extremely elevated, clinical range | Extremely elevated, clinical range | Yes | Deteriorated |  | Functional | Clinical | No | Unchanged |
| Waitlist P11 | Extremely elevated, clinical range | Functional | Yes | Recovered |  | Functional | Functional | No | Unchanged |
| Waitlist P12 | Clinical range | Clinical range | No | Unchanged |  | Clinical | Clinical | No | Unchanged |
| Waitlist P13 | Elevated, functional range | Functional | No | Unchanged |  | Functional | Functional | No | Unchanged |
| Waitlist P14 | Elevated, functional range | Functional | No | Unchanged |  | Functional | Functional | No | Unchanged |
| Waitlist P15 | Clinical range | Elevated, functional range | No | Unchanged |  | Functional | Functional | No | Unchanged |
| Waitlist P16 | Extremely elevated, clinical range | Extremely elevated, clinical range | Yes | Deteriorated |  | Clinical | Clinical | No | Unchanged |
| Waitlist P17 | Extremely elevated, clinical range | Elevated, clinical range | No | Unchanged |  | Functional | Functional | No | Unchanged |
| Waitlist P18 | Extremely elevated, clinical range | Extremely elevated, clinical range | Yes | Deteriorated |  | Clinical | Functional | No | Unchanged |
| Waitlist P19 | Functional | Functional | No | Unchanged |  | Functional | Functional | No | Unchanged |
| Waitlist P20 | Extremely elevated, clinical range | Extremely elevated, clinical range | Yes | Improved |  | Functional | Functional | No | Unchanged |
| Waitlist P21 | Elevated, functional range | Elevated, clinical range | Yes | Deteriorated |  | Functional | Functional | No | Unchanged |
|  |  |  |  |  |  |  |  |  |  |
|  | RCADS Anxiety | | | |  | RCADS Depression | | | |
| Group | Pre-Treatment Score Classification | Post-Treatment Score Classification | Reliable Change Achieved | Clinically Significant Classification |  | Pre-Treatment Score Classification | Post-Treatment Score Classification | Reliable Change Achieved | Clinically Significant Classification |
| ICBT-P P1 | Functional | Functional | No | Unchanged |  | Functional | Functional | No | Unchanged |
| ICBT-P P2 | Clinical | Functional | Yes | Recovered |  | Functional | Functional | No | Unchanged |
| ICBT-P P3 | Clinical | Subclinical | No | Unchanged |  | Clinical | Clinical | No | Unchanged |
| ICBT-P P4 | Clinical | Functional | Yes | Recovered |  | Clinical | Functional | Yes | Recovered |
| ICBT-P P5 | Functional | Functional | Yes | Recovered |  | Clinical | Functional | No | Unchanged |
| ICBT-P P6 | Clinical | Functional | Yes | Recovered |  | Subclinical | Functional | No | Unchanged |
| ICBT-P P7 | Clinical | Subclinical | Yes | Improved |  | Clinical | Functional | Yes | Recovered |
|  |  |  |  |  |  |  |  |  |  |
| Waitlist P8 | Functional | Functional | No | Unchanged |  | Functional | Functional | No | Unchanged |
| Waitlist P9 | Functional | Subclinical | No | Unchanged |  | Subclinical | Subclinical | No | Unchanged |
| Waitlist P10 | Functional | Clinical | No | Unchanged |  | Functional | Functional | No | Unchanged |
| Waitlist P11 | Subclinical | Clinical | Yes | Deteriorated |  | Subclinical | Functional | No | Unchanged |
| Waitlist P12 | Subclinical | Functional | No | Unchanged |  | Functional | Functional | No | Unchanged |
| Waitlist P13 | Subclinical | Subclinical | No | Unchanged |  | Clinical | Clinical | No | Unchanged |
| Waitlist P14 | Functional | Functional | No | Unchanged |  | Subclinical | Functional | No | Unchanged |
| Waitlist P15 | Clinical | Clinical | No | Unchanged |  | Subclinical | Clinical | No | Unchanged |
| Waitlist P16 | Clinical | Clinical | Yes | Deteriorated |  | Clinical | Clinical | Yes | Deteriorated |
| Waitlist P17 | Functional | Subclinical | Yes | Deteriorated |  | Functional | Functional | Yes | Deteriorated |
| Waitlist P18 | Clinical | Clinical | No | Unchanged |  | Clinical | Clinical | No | Unchanged |
| Waitlist P19 | Functional | Functional | No | Unchanged |  | Functional | Functional | No | Unchanged |
| Waitlist P20 | Clinical | Subclinical | Yes | Improved |  | Subclinical | Subclinical | No | Unchanged |
| Waitlist P21 | Functional | Functional | No | Unchanged |  | Functional | Functional | No | Unchanged |
|  |  |  |  |  |  |  |  |  |  |
| *Note.* *N* = 21 (ICBT-P *n* = 7, Waitlist control *n* = 14). P1 - P21 = Participant number; ICBT-P = Internet-Based Cognitive Behaviour Therapy for Perfectionism; EDE-Q Global = Global score on the Eating Disorder Examination Questionnaire; CPQ = 12-item total on the Clinical Perfectionism Questionnaire (CPQ); RCADS Anxiety = Revised Child Anxiety and Depression Scales (RCADS) T-score on the total subscales for anxiety; RCADS Depression = T-score on the total RCADS Depression subscale. | | | | | | | | | |
